# The Role of Fruit by-Products as Bioactive Compounds for Intestinal Health

**DOI:** 10.3390/foods9111716

**Published:** 2020-11-22

**Authors:** Mohamed Aymen Chaouch, Stefania Benvenuti

**Affiliations:** Department of Life Sciences, University of Modena and Reggio Emilia, Via G. Campi 103, 41125 Modena, Italy; stefania.benvenuti@unimore.it

**Keywords:** fruit by-products, bioactive compounds, dietary fibres, intestinal function, food, gut

## Abstract

The fruit processing industry generates large amounts of wastes (pomace, seeds, peels) that causes negative environmental impact with considerable treatment expenses. Nevertheless, various studies demonstrated that these by-products are still rich in bioactive compounds, especially dietary fibres and phenolic compounds, thus leading to significant chemical, physical and biological properties. These characteristics make fruits by-products a good source for new supplements in food products having important effect on intestinal function. Thus, the aim of this review is to evaluate the different bioactive compounds isolated from fruit by-products and to analyse their application in various formulations for the food and nutraceutical industries. In consideration of the biological properties of these compounds, their role in the functioning and action on intestine and gut flora was discussed.

## 1. Introduction

The significant progress made in the field of chemistry, biology and agriculture has enabled better exploitation of agro-resources. Food is not seen as a simple nourishment, but its beneficial or harmful effects for the organism, and its role in the prevention of cardiovascular and metabolic pathologies are taken into consideration. No less important for the consumer is the impact on the environment of food production, both in terms of energy and human resources used, and of residues generated by agronomic practices, transformation and conservation. In this context the recovery of waste from agro-resource processing industries is considered of notable importance not only for economic matters but also for environmental sustainability, and offers new opportunities for economic development in many sectors. Thus, the economic burden of recycling waste becomes the production of high value-added co-products intended for resale to the biomedical, food, cosmetics, chemistry and design packaging materials industries [1,2]. The use of agro-resources and their co-products is motivated by their abundance, their renewable nature, their biodegradability and the added value which must justify any industrial development. Indeed, agro-resources could offer a new source of raw materials in many fields.

Even if the current globalization of the market ensures the availability of fresh fruit throughout the year, a large part is subjected to different transformation processes in order to obtain new products satisfying the different current demands of consumers. Thus, it has been reported that about a third of the edible portion of food intended for human consumption is lost or wasted along the food chain, from initial production to final consumption [3,4]. The large quantity of residues produced by the food industry, in addition to being a great loss of valuable materials, also raises serious management problems from an economic and ecological point of view. To address these economic and ecological problems, the recovery of residues represents a promising solution to absorb millions of tons of waste material. The valorization of by-products from the food industry gives a second life to co-products and limits the use of conventional energy sources [5].

Depending on the nature of the co-products to be recovered, several techniques have been developed. The transformation of co-products into biofuels and biosolvents represents a very promising economic and ecological recovery [6,7]. The integration of co-products into the animal feed market also means a promising recovery technique [4]. Fruit and vegetable co-products have been valued in the field of functional foods because of their health interests [8,9].

## 2. Bioactive Compounds from Fruit by-Products

Several researches have proven the presence of a wide range of bioactive compounds in various fruit industrial by-products which are essentially pomace, peels and seed fractions. These compounds consist mainly of carbohydrates (pectin, cellulose, hemicellulose…), secondary metabolites (phenolics, glycosides, alkaloids, gums, mucilage and volatile oils), lipids and proteins. Generally, seeds are rich in polyphenols and bioactive lipids whereas peels are considered as a rich source of dietary fibres [10,11]. Bioactive compounds are present in fruit by-products with various concentrations and combinations. These differences could be mainly related to the fruit variety, geographic location, maturation stage, as well as extraction parameters (solvent, extraction ratio, time and temperature) [9].

Several extraction techniques were carried out for the isolation of bioactive compounds from fruit by-products. For example, solid state fermentation (SSF) was applied for the extraction of polyphenolic antioxidants from grape skin [12] and bagasse [13] and for the isolation of ellagic acid from pomegranate Husks [14]. Citric acid was isolated from banana peel by SSF using *Aspergillus niger* [15], while pectin and limonene were extracted from orange peel through enzymatic and chemical hydrolysis [16]. Mango peels were subjected to autoclave treatment for the extraction of pectin and polyphenols [17]. The other processes that we can cite are ultrasound treatment (fatty acids and tocopherols from watermelon seeds) [18], microwave-assisted extraction (phenolic compounds from pitaya fruit peels) [19], steam explosion (limonene from orange peel) [20] and classic extraction with ethanol (phenolic antioxidants from avocado peel) [21] or n-hexane (antioxidant oils from melon seeds and carotenoids and anthocyanins from papaya peel) [22,23]. Another modern method for the isolation of bioactive compounds from fruit by-products is sub- and supercritical fluid extractions. This method was applied for the extraction of phenolic compounds, flavonoids, carotenoids, pectin, reducing sugars, lipids and proteins from several by-products such as citrus peels and pomace, pomegranate peels, apple pomace and seeds, grape pomace and seeds [24].

Plant bioactive metabolites exert pleiotropic effects by the modulation of multiple metabolic pathways through a variety of molecular targets. Previous studies revealed that dietary phenolic compounds displayed a pleiotropic behavior on key proteins, thus presenting beneficial effects in several chronic disorders which are related to oxidative stress, inflammation and aging. This is strongly related to the wide range of biological activities like antioxidant, antimicrobial, anti-inflammatory, anti-allergenic, anticancer and cardioprotective activities. Thus, it has been reported that natural phenolic compounds and their metabolites exerted significant effects on the main metabolic pathways involved in energy metabolism (the AMP-protein kinase AMPK and the mammalian target of rapamycin mTOR are the main regulators), as well as inflammatory response and aging (main regulators are the nuclear factor-erythroid 2 p45-related factor 2 (Nrf2) and sirtuins) [25,26]. For example, Marín-Aguila et al. (2013) described some nutraceutical compounds targeting AMPK pathways in cancer, cardiovascular disease, type 2 diabetes mellitus and neurodegenerative disease including phenolic acids, anthocyanins, stilbene, flavone, flavonol, alkaloids, lignan [26]. Thus, bioactive compounds isolated from fruit by-products showed a significant effect as bioingredients in functional foods as well as nutraceuticals in pharmaceutical and medicinal recipes. This was mainly due to their antioxidant, anti-inflammatory, antimicrobial, anti-allergenic, antithrombotic, anti-atherogenic, cardioprotective and vasodilatory capacities [11].

### 2.1. Phenolic Compounds

Various phenolic compounds were isolated such as hydroxybenzoic and hydroxycinnamic acids, flavonoids (flavonols, flavanones, flavones, flavanonols, isoflavones, flavanols, and anthocyanidins), lignans and stilbenes (Table 1). Many fruit wastes showed highest phenolic content, especially grape, pomegranate, apple and citrus varieties (orange, lemon).

Indeed, the interest accorded to these compounds was mainly related to their capacity to scavenge free radicals and to regulate the generation of free radicals in vivo, thus ensuring the prevention of oxidation reactions in food and cell damage. These characteristics allows them to replace synthetic preservatives [10].

For example, an important oxidative stability was found in the oil extracted from date seeds due to the higher phenolic content (about 19 mg GAE/g dry weight). This ensures the use of date seed oil as natural additive to other vegetable oils in order to improve its heat stability [27,28]. In addition, big amounts of seeds and peels residues are generated by the citrus industry. These residues constitute about 50% of the total fruit and are an important source of phenolic compounds. Also, it has been reported that the peels of many fruits, such as apples, peaches, pears, banana and pomegranate, have been found to contain highest content in phenolics than the edible portions [29].

### 2.2. Dietary Fibres (DFs)

Over the last decades, there has been an increasing trend to recover dietary fibre (DF) compounds from industrial by-products. These compounds refer essentially to the sum of non-starch polysaccharides and lignin. Thus, it has been reported that fruit by-products are mainly composed of cellulose, hemicellulose, pectin, gums and lignin [10].

Table 2 illustrates some DF compounds isolated from various fruit by-products.

DF compounds can be obtained from the by-products of various food processing industry, such as the beverage, canning and juice industries. This latter probably produces the most important amounts of by-products, composed mainly by pomace and peels [60].

From a general aspect, the interest given to DF is strongly associated to their significant role in decreasing many health disorders. Cellulose, hemicellulose and lignin are well-known for water absorption and intestinal regulation, whereas pectin and gums showed important effects in cholesterol reducing and glucose regulation [61].

The data reported refer to the dry weight, but the high amount of water in these by-products must be considered. The freeze-drying or spray-dry operations to remove water have to be considered because of their cost rather than preparing a concentrate with less energy consumption for the preparation.

Moreover, DFs showed widespread use in the food industry when they are incorporated into bakery products by enhancing the digestion, prolonging the freshness and retaining more water. They also improve the texture and provide a desirable resistance to melting of ice cream [61].

### 2.3. Proteins and Peptides

Proteins are important biomolecules for a good function of the human body, particularly to form muscles [29]. Thus, it has been reported that many health diseases are strongly related to protein deficiency such as Kwashiorkor, Marasmus (energy deficiency), mental disorders, organ failure, oedema and weakness immune system [69].

In more recent diets, especially aimed at athletes or for diseases related to diabetes and the cardiovascular system, increased protein intake plays an important role. It is also to be considered that a greater ecological awareness leads people more and more often to limit the consumption of meat, if not actually not to use it as is the case for vegan people.

The use of plants, fungi and their extracts as meat substitutes have become increasingly important in nutrition and satisfies the request for proteins and amino acids, essential for the regular human metabolism. Indeed, fruit by-products have been reported to be an important source of proteins and peptides (Table 3). These latter are generally obtained by the hydrolysis of proteins [10].

In comparison with vegetables, it has been reported that the oil isolated from hempseeds is mainly composed of polyunsaturated fatty acids, particularly linoleic (ω-6) and α-linolenic (ω-3) acids, whereas globulin (edestin) and albumin were found to be the major proteins [77]. The by-product resulted from canola oil extraction (Canola meal) is very rich in proteins (up to 50%), whereas canola seeds contain about 26% of protein. The protein content of canola meal, which consists mainly of napin and cruciferin, permits its use for human food and animal feed [78].

Marcet et al. reported that raw rice bran and raw soybean contained high amounts of peptides, 75% and 50% of the total protein content, respectively, whereas the amount of recovered amino acids from these two by-products was 5% of the total protein content. Peptides were also isolated from soy pulp with a percentage of 35% from the total dry matter [79].

Leuk leaves showed high crude protein content (19.4% on dry matter basis) with a total amino acid content of 14.1% (mainly Leu and Lys, 11.6 and 8.2 mg/g dry matter, respectively), while protein content in parsley was 17.0% from dry matter in which the percentage of essential amino acid was 40%. The most abundant ones were Leu and Lys, 12.4 and 8.3 mg/g dry matter, respectively [80].

### 2.4. Lipids

Lipids, water-insoluble molecules, are in essential components for the human organism. They play an important physiological and biochemical rolein the function of the human body, such as energy storage (fats and oils), structural components of biological membranes (phospholipids and sterols), electron carriers, enzyme cofactors, light-absorbing pigments, hydrophobic anchors for proteins and emulsifying agents in the digestive tract [81,82]. Besides their important nutritional role in the human diet, lipids are also exploited as food ingredients, thus improving texture, mouthfeel and flavour of new formulations [83]. Indeed, due the increasing demand for vegetable oils, the interest was oriented to the possibility of exploiting new oil sources with higher amount of polyunsaturated fatty acids. In this context, fruit by-products, particularly seeds, have been reported to be a potential alternative for lipids production. Several fatty acids were isolated from various fruit by-products such as linoleic acid, linolenic acid, palmitic acid, palmitoleic acid, oleic acid, lauric acid, myristic acid, stearic acid, lignoceric acid, arachidic acid, erucic acid [83].

Table 4 present the lipid content in various fruit by-products.

Apple seeds were reported to contain significant amount of lipids (277 g oil/kg apple seeds), in which unsaturated fatty acids were the predominant (89 g/100 g oil). These lipids are mainly linoleic acid (51.2 g/100 g oil), whereas others are palmitic (10.5 g/100 g oil), linolenic (5.6 g/100 g oil), stearic (4.3 g/100 g oil) and oleic acids (4.1 g/100 g oil) [89]. Wild and cultivated berries seeds are also an important source of lipids (14% to 18% of dry matter). Their rich composition in α-linoleic acid and their high content of α- and γ-tocopherols allows them to be beneficial for balancing diet fatty acid composition and skin regeneration [90,91]. Plum seeds are rich in sterol esters and n-3 PUFA (omega-3) [90], whereas the main lipids in passion fruit seeds are stearic acid, palmitic acid, oleic acid and linoleic acid [92].

## 3. Fruit by-Products and Intestinal Function

In the light of all above, we can assert that fruit by-products are a good source of bioactive compounds. In fact, from economic and environmental point of view, it is very important to proceed to the valorization of these wastes in many fields.

The most popular process for fruit by-products management is their incorporation into livestock feeds. In this context, numerous fruit by-products demonstrated the ability to be incorporated into animal feed because of their rich composition in proteins, digestible fiber, soluble sugars, vitamins and minerals. In addition, the majority of these by-products don’t present any adverse impacts on the productive and reproductive performance of animals. However, some by-products need some precautions when applying them in animal feed due to various factors such as limited shelf life (high moisture content), presence of pathogens agents or antinutrient metabolites [93]. Among the widely used fruit by-products for animal feed we can cite citrus pulp, [94] grape pomace [94], mango seeds [95], banana peels and leaves [96], apple waste [8,96], grape waste [96], pomegranate waste [96], banana peels [97], passion fruits peels [97] and bilberry pomace [8].

Beside their use as animal feed or to reduce methane production, the interest was oriented over the last decades to the application of fruit by-products in the food, cosmetic and pharmaceutical industries [48]. Thus, it has been reported that the incorporation of some fruit by-products into food products improves the quality of these products. This improvement was affirmed by the significant impact on the sensory evaluation (texture, taste, odor, color, overall acceptability…) of the new formulations (Table 5) [82].

Bioactive compounds from fruit by-products find several applications in the different fields of food processing industry. They could be considered as additives in food products, thus promoting the preservation and the enhancement of the quality, as well as the prevention of food oxidation and pathogenic microorganisms growth. In this context, pomegranate and grape seed extracts were reported to be good natural additives in foods and food packaging industry, due to their significant antioxidant and antimicrobial properties [98]. Another application of fruit by-products in the food processing industry is their use as packaging materials which is strongly related to the oxygen-impermeable properties. Various studies found promising results when incorporating fruit by-products into edible films that could be used as food packaging materials [99]. Other researches revealed also that fruit by-products can be also used as a reserve of wheat flour (example of berry pomace) [100], as a supplement in cakes and cookies (example of sour cherry pomace) [101] or in fermented beverages production (example of blueberry bagasse) [102]. Fruit by-products are now considered as value-added supplements for foods, due to their high content in bioactive compounds, especially dietary fiber. Indeed, their incorporation into foods leads to several beneficial effects such as the improvement of water and oil retention and the enhancement of oxidation stability and emulsification properties, as well as their consideration as non-caloric bulking agents [103].

According to the European Food Safety Agency (EFSA) and the European Commission (EC), some rules should be respected when talking about foods. For example, food additives are compounds which are added to a food product to achieve a specific technological aim, but they are not ingested as a food product. Indeed, according to EFSA, while accomplishing a specific technological goal (satisfaction of specific dietary requirement, Improvement of sensory quality, facility of production, packaging, transport and storage), a food additive should not present any safety trouble for consumers health and must attend specific purity criteria [104,105,106]. Thus, it has been reported that some additives, which are currently allowed by EC, are present in fruit by-products including anthocyanins (E163) (example of grape by-products) [107] and chlorophylls (E140) (principally in green leaves and mango peels) [108]. The different types of food additives and their functions as cited by European Food Safety Agency (EFSA) and European Commission (EC) were mentioned by Faustino et al. (2019) [109].

The gut microbiota plays an important role in the function of human gastrointestinal tract thought its interaction with food components and host cells. Thus, it has been reported that a balanced gut microbiota was strongly related to the adjustment of the immune system and antioxidant defense, while microbiota disorders leads to several health diseases such as rheumatoid arthritis, colorectal cancer, obesity, diabetes, irritable bowel disease, inflammatory bowel disease and cardiovascular disease [114,115].

Recently, gut microbiota has been investigated as a possible environmental factor influencing obesity through its role in fat storage. The intestinal microbiota contributes to the absorption by the host of carbohydrates and lipids and regulates the storage of fat [116]. In addition, it was observed that it occurs a change in the composition of the gut microbiota (dysbiosis) for people suffering from colorectal cancer or chronic inflammatory bowel disease [117,118]. Recent researches revealed that there is an accordance between autistic disorder and gastrointestinal disorders. Preliminary studies also showed that there is a form of dysbiosis in autistic children with a decrease in *Akkermansia muciniphila* and *Bifidobacterium* spp. in the stools of these patients [119,120].

Besides that, the microbiota plays an essential role in the maturation of the immune system and the development of its functions. This role is suggested by the many abnormalities observed in axenic mice: hypoplasia of Peyer’s patches, decreased number of intraepithelial lymphocytes, low production of serum immunoglobulins and cytokines. In addition, the spleen and lymph nodes of axenic mice show areas of atrophied lymphocytes. All of these abnormalities disappeared within a few weeks after inoculation of the microbiota of conventional mice into axenic mice [121].

Diet is a subject of very important interest in research programs because of its potential to modulate the intestinal microbiota of the host, whether beneficial or harmful. Eating habits have a significant impact on the composition of the intestinal microbiota, especially in the first years of life [122].

Dietary fiber intake can benefit people having a wide variety of metabolic syndromes and gastrointestinal disorders [123,124]. High fiber intake has been reported to be beneficial for patients with diabetes, high cholesterol, hypertriglyceridemia, obesity or hypertension [124,125]. It has also been reported that individuals with high fiber intake are less susceptible to the development of cardiovascular disease or colon cancer [126]. Likewise, diets with a high intake of fats and proteins, but with a low fiber content, are associated with a higher risk of developing colon cancer, unlike vegetarian or oriental diets with higher fiber intakes [127,128]. In addition, Japanese people who adopt a western diet are more likely to develop colon cancer [129].

DFs are the main source of carbon and energy for colonic microbes. The major components of DFs are non-starch polysaccharides (20–45% of the dry matter supplied to the colon), simple sugars (10%), oligosaccharides (10%) and starch and starch hydrolysis products (about 8% of dry matter) [122]. Furthermore, the proximal colon received daily about 5–10 g of lipid and 5–15 g of protein, mainly of dietary origin, in addition to some other minor components like polyphenols, lignin, tannins, catechins and micronutrients [122]. In the other hand, it has been also reported that the major part (about 90%) of 1 g/day of dietary polyphenols escapes absorption and digestion in the small intestine and persists in the colon. Then, by contact with the gut microbiota, they become like compounds for microbial production of small phenolic acids and SCFA or, thus having effect on species composition and their metabolic activity [130].

DFs were reported to have both preventative and therapeutic impact for various bowel intestine-related diseases such as obesity, type II diabetes and cardiovascular diseases [131]. They are fermented by the colonic microbiota to produce other metabolites like organic acids that supply energy for other bacteria, bowel epithelium and peripheral tissues [132]. DFs preserve bowel health by increasing digesta mass, which leads to the reduction of intracolonic pressure, dilution of toxins, and decrease of transit time, as well as an augmentation of defecation frequency. This increase was mainly due to the physical properties and the ability to adsorb water, especially of incompletely fermented fiber (insoluble non-starch polysaccharides, like cellulose) [132]. An increase in the fecal mass could be also made by DFs thought the enhancement of fermentation, thus conducting to bacterial proliferation and biomass increase [133].

Dietary polyphenols were also reported to be beneficial for the prevention of health from several diseases like cardiovascular disease and cancer due to their significant antioxidant or anti-inflammatory effects [122]. Generally, polyphenols are found in plant foods as a bound form (mostly as glycosides). The major part of them undergoes a metabolization process by gut microbiota, thus forming aglycones [134]. Flavonoid quercetin, flavonols, caffeic acid, (+)-catechin, (−)-epicatechin, resveratrol, ellagitannin and anthocyanins were reported to have significant impact on the modulation of the gut microecology [134].

Nowadays, it is well-known that obesity could be treated using diet with moderate amounts of protein, thus reducing health disorders associated with obesity. Moreover, dietary proteins also showed significant effects on gut health, particularly when combined with carbohydrates [122]. They are also the main source of nitrogen for colonic microbial growth [122]. Furthermore, fatty acid rich-diets showed positive effect on immune function, cholesterol and triglycerides levels, blood pressure and cardiovascular function [135,136].

Although cereal grains and their by-products are the most known source of DFs, it has been reported that fruit by-products could be an alternative source of DF because of their high content in DFs. Indeed, DFs are considered as important elements for the good function of human body, particularly for the improvement of the growth of beneficial of hind gut microbiota, the satiety and attenuation of constipation and the regulation of glucose and lipid levels, thus reducing the risk of coronary heart and cancer diseases [137].

The gastroprotective effect of several fruit by-products was studied, such as orange bagasse [138], passion fruit peels [138], grape pomace [139], grape peels [140], grape seed [141], pomegranate by-products [142,143,144], orange peels [145], apple peels [146], apple pomace [147,148] (Table 6).

In this context, Abboud et al. (2019) studied the gastroprotective effect of yellow passion fruit peel, which is an unexploited by-product from the juice industry in Brazil. Soluble dietary fibres (SDF) isolated from this by-product showed significant gastroprotective effects on animals following oral and intraperitoneal digestion. This effect was manifested by the decrease of gastric ulcer lesions induced by ethanol and the prevention of the exhaustion of gastric wall mucus and reduced glutathione (GSH) levels. Thus, oral pre-treatment with SDF reduced significantly the ethanol-induced gastric lesions in 72.25%, 79.23% and 87.17% by injection of 0.1, 1 and 10 mg/kg, respectively. The same doses were able to avoid the depletion of GSH levels in 52.20%, 41.91% and 50.33% and the gastric wall mucus in 26.32%, 25.03% and 31.00%. Moreover, the treatment by intraperitoneal route with SDF (1 mg/kg) reduced the gastric lesion area in 72.56% and prevented the depletion gastric wall mucus in 21.24% and the GSH levels in 40.81% [149].

Another research conducted by Athaydes et al. (2019) focused on the treatment of gastric disorder by avocado seeds, which is a by-product generally discarded as waste. The total indomethacin-induced gastric lesion area was significantly reduced after the treatment with avocado seeds (92% of protection). Results showed also that an injection of 75 mg/kg of ethyl acetate extract from avocado seeds increased mucus production by the stomach mucosal cells of about 2.38 ± 0.55 times compared to the control, which forms a mucoid layer, thus promoting tissue repair and accelerating epithelial recovery. The authors suggested that phenolic compounds present in this by-product are the main responsible on this significant effect [150].

Pomegranate peels extract has been reported to possess significant effects on stomach gastric mucosa (at a dose of 500 mg/kg), mainly manifested by the protective role against ethanol induced gastric ulcer in rats. This anti-ulcer impact was asserted by the high levels of TGFβ1 in serum and the decrease of ulcer lesion areas in the stomach layer. This significant antiulcer effect might be associated to existence of several bioactive compounds such as anthocyanins, anthocyanidins, punicic acid, ellagic acid, flavonoids and ellagitannins [152].

A significant anti-ulcer effect was also mentioned for the aqueous extract from orange (*Citrus sinensis*) peels. This effect was shown through the correction of the enhanced gastric lipid peroxidation and the reduction of TNF-α (tumor necrosis factor alpha) production, as well as the reduction of gastric DNA fragmentation and gastric COX-2 (cyclooxygenase-2) expression. Authors suggested that this beneficial effect was related to the capacity in chelating free iron and scavenging H_2_O_2_, thus leading to calcium homeostasis. In addition, the pre-treatment with Citrus sinensis extract ensure protection against the overload of cells of the gastric and duodenal mucosa caused by the sub-acute exposure of ethanol [153].

Polyphenol-rich extract from apple peels exhibited also an important inhibitory effect against *Helicobacter pylori* attachment, by avoiding of initial steps in the *Helicobacter pylori* colonization process and suppressing inflammation. Oral ingestion with doses of 150 and 300 mg/kg/day was reported to be good enough to avert the initial attachment of *Helicobacter pylori* to the antral mucosa which causes an inflammatory damage. The decrease in this damage by apple peel phenolic extract was essentially associated to its important antioxidant and anti-VacA (vacuolating bacterial toxin) properties. Thus, it has been reported that gastric *Helicobacter pylori* infection, which affects about half of the world’s population, is the main cause of various gastrointestinal disorders like gastric carcinoma and duodenal ulcer [154]. On the other hand, Mousa et al. (2012) asserted that apple pomace pectin could be a potential gastroprotective and hypolipidemic drug due to its significant content on citric and galacturonic acids. The gastroprotective effect against indomethacin induced ulcer was reported by preventing the formation of gastric mucosa lesions, lining the surface of the stomach and improving the resistance of the gastroduoden mucosa [147].

Grape by-products have been also studied for their effects on intestinal function. For example, Bhardwaj et al. (2018) investigated the impact of proanthocyanidins from grape seeds on different experimental models of gastric ulcers in rats. A 100 mg/kg dose of grape seed proanthocyanidin showed better treatment of gastric ulcers with fewer side effects. This effect was observed by the significant decrease in ulcer index and gastric acidity in pylorus ligation models, as well as the reduction in MDA (malondialdehyde) levels [141].

Sen et al. (2014) reported that pectin isolated by hot water-acid extraction from various fruit by-products (apple *Malus domestica*, lemon *Citrus limon* and orange *Citrus sinensis*) showed an important role in supporting the survival and the development of the probiotics in the gastrointestinal tract [157].

Fibers isolated from orange bagasse and passion fruit peels were reported to have significant impact on the modulation of the gut microbiota. They could be considered as an energy source for bacteria located in the distal colon. Thus, the negligible production of lactate and succinate permit the slow fermentation, which allows protection against colon cancer [138].

The effect of acerola by-product on the gut microbiota metabolism was also studied. The treatment with acerola by-product was found to have positive effect since a decrease of the NH_4_^+^ amount in the ascending colon was observed, as well as an increase in short-chain fatty acids (SCFAs) in the three regions of colon. This important effect was mainly due to high content in fibre and phenolic compounds in acerola by-product, which are responsible for the high antioxidant activity [155].

Another research conducted by Batista et al. revealed that by-products from acerola (*Malpighia emarginata* D.C.), cashew (*Anacardium occidentale* L.) and guava (*Psidium guajava* L.) fruits could be considered as protective compounds for intestinal health against the harmful effects induced by dyslipidaemic diet. Their role is the protection of colon and liver from tissue damage. This important role was experimentally assured manifested by several parameters such the decrease of faecal pH, serum lipid levels, liver fat and visceral fat, as well as the increase of faecal moisture and faecal fat excretion in dyslipidaemic female rats [156].

The consumption of insoluble dietary fiber from citrus by-products was strongly linked to intestinal motility by delaying the gastric emptying and extending the effective unstirred layer, which makes the absorption process in the small intestine slower thus giving an extended feeling of fullness. Several factors have been reported to assert the protective effect of fibers against gastrointestinal diseases including the decrease in intracolonic pressure and the transit time and the increase in stool weight. In the other hand, soluble dietary fibers from citrus by-products were considered as important compounds in reducing risks of coronary heart diseases by lowering triacylglycerol concentrations and enhancing insulin sensitivity. This was asserted through the delaying of the absorption process of macronutrients, especially carbohydrates and fat [158].

## 4. Conclusions

In conclusion, we can state that the large amounts of wastes generated by fruit processing industry represent a great origin of bioactive compounds having significant potential benefits for various applications in several industries. These by-products contain high contents of dietary fibres, polyphenols, lipids and proteins as described in this review, which makes them a considerable source of food additives. The latter could be integrated in several food industry sections like bakery products, dairy products, biscuits, cakes.

Nevertheless, several fruits by-products have been reported to exhibit potential good effect on the intestinal function. Treatment with dietary fibres, polyphenols or anthocyanins from several fruit by-products showed a significant effect on the improvement of gut health and the treatment of gastric disorders.

## Figures and Tables

**Table 1:** Content of phenolic compounds in some fruit by-products.

Fruit by-Product	TPC	Phenolic Compounds	References
Pomegranate peel	139.4 *	Punicalagin A, punicalagin B, catechin, gallic acid, ellagic acid	[30,31,32]
	420.6 ***		
Pomegranate pomace	134.8 **	Gallic acid, catechin, ellagic acid, rosmarinic acid, hesperidin, p-coumaric acid, chlorogenic acid	[33,34]
Rowanberry pomace	167.4 ****	Cyanidin, Chlorogenic acid, quercetin, kaempferol	[35,36]
Apple pomace	13.8 *	Hydroxycinnamic acids, Hydroxycinnamates, phloretin glycosides, quercetin glycosides, catechins, procyanidins	[37,38]
Apple peel	34.3 *	Gallic acid, caffeic acid, vanillic acid, catechin, epicatechin gallate, chlorogenic acids, phloridzin, rutin	[38,39]
Banana peel	29.2 *	Epicatechin, rutin, hydroxybenzoic acid, myricetin, ferulic acid, chlorogenic acid, gallic acid	[40]
Date by-products	4.4 *	Quercetin, luteolin, apigenin, chrysoeriol, kaempferol, isorhamnetin, malonyl derivatives	[41,42]
Elderberry pomace	4.7 *	Cyanidin, rutin, oleanolic acid, ursolic acid, linoleic acid	[43,44]
Grape juice by-product	23.4 *	Benzoic and hydroxycinnamic derivatives, catechins, flavanols, anthocyanins, tannins, proanthocyanidins	[45,46]
Grape pomace	142.1 *	Phenolic acids (ferulic, p-coumaric, caffeic, gallic, vanillic, p-hydroxybenzoic), flavanols (proanthocyanidins), flavonols (kaempferol, quercetin, myricetin), stilbenes (resveratrol, piceid, astringin), anthocyanins	[47,48]
Grape seed	74.0 *	Gallic acid, caftaric acid, catechin, epicatechin, epicatechin gallate, procyanidins, resveratrol	[48,49,50]
Mango kernel	72.1 *	Gallates, gallotannins, gallic acid, ellagic acid and its derivatives	[48,51]
Orange by-product	4.21 *	Caffeic acid, Ferulic acid, p-Coumaric acid, Eriocitrin, Narirutin, Hesperidin, Neohesperidin	[52,53]
Orange peel	65.7 *	Caffeic acid, p-coumaric acid, naringin, kaempferol, neohesperidin, rutin	[54]
Orange pulp	22.3 *	Flavonone (Eriocitrin, Narirutin, Hesperidin, Didymin…), Flavone (Quercitrin, Nobiletin…), Kaemperol, Benzoic acids, Cinnamic acids, Chlorogenic acid,	[55]
Lemon peel	49.8 *	Caffeic acid, Coumaric acid, Ferulic acid, Sinapic acid	[56,57]
Passion fruit by-products	3.84 *	p-coumaric acid, Epicatechin	[52,58]
Guava by-product	19.9 *	Resveratrol, coumarin	[23]
Cherry by-product	91.3 *	Flavonoids, anthocyanidins, stilbenes, resveratrol, quercetin, gallic acid	[59]

* mg gallic acid eq./g extract DW; ** mg gallic acid eq./g liquid extract; *** mg tannic acid eq./g extract DW; **** mg catechin eq./g extract DW.

**Table 2:** Dietary fibre content in some fruit by-products.

Fruit by-Product	TDF (g/100 DW)	References
Apple Pomace	45.0	[62]
Apple Peel	43.9	[63]
Apple by product	75.8	[64]
Banana Peel	49.6	[40]
Orange Pomace	63.8	[65]
Orange Peel	48.7	[66]
Orange by-product	58.2	[52]
Passion fruit by-product	64.2	[52]
Guava by-product	89.8	[52]
Date seeds	73.5	[67]
Grape fruit by-product	67.2	[64]
Apricot by-product	72.3	[64]
Pomegranate Peel	56.2	[68]
Pomegranate pomace	43.5	[34]

**Table 3:** Protein content in some fruit by-products.

Fruit by-Product	Protein (%)	References
Orange by-product	5.2	[52]
Passion fruit by-product	12.6	[52]
Guava by-product	2.1	[52]
Date seeds	6.0	[67]
Pomegranate peels	12.9	[70]
Pomegranate pomace	11.1	[34]
Apple Pomace	4.8	[71]
Apple Peel	3.2	[63]
Mango peel	4.3	[72]
Banana Peel	7.0	[73]
Orange juice by-product	18.9	[70]
Orange Pomace	9.8	[74]
Orange Peel	6.8	[75]
Citrus peel	4.5	[76]
Grape fruit by-product	5.8	[13]

**Table 4:** Lipid content in some fruit by-products.

Fruit by-Product	Lipid (%)	References
Orange juice by-product	8.4	[70]
Pomegranate peel	3.2	[70]
Pomegranate by-product	4.0	[84]
Passion fruit by-product	8.0	[52]
Guava by-product	1.2	[52]
Apple Pomace	4.2	[85]
Apple Peel	10.1	[63]
Berry pomace	20.2	[36]
Grape fruit pomace	8.5	[86]
Banana Peel	2.0	[87]
Date seeds	8.8	[88]

**Table 5:** Application of some fruit by-products into food products.

Fruit by-Product	Food Products	Effects	References
Apple pomace	Bakery products, cakes, cookies, meat products, yoghurt, jams, juice	- Source of dietary fibre and polyphenols - Enhance nutritional value - Natural stabilizer and texturizer	[85]
Grape pomace and seeds	Bakery products, yoghurt, Meat product	- Dietary fiber supplement - Modify the formulation and extend shelf life of meat - Reduction of meat lipid oxidation	[110]
Banana peel	Bakery products, Pasta, Confectionaries	- High gluten bread - High ash, dietary fibre and total phenolic content - Increase of viscosity, volume, gumminess and odour acceptance - Increase in foaming stability and - overall acceptability	[111,112]
Mango by-product	Bakery products, biscuits	- High content of dietary fiber and polyphenols - Increase of breaking strength, weight and density - Good sensory acceptability	[60,111]
Orange by-product	Biscuits, sausage, Fermented milk, Ice cream, Pasta products	- Wheat flour or starch substitute - Improve nutritional value - Reduce the caloric value - Good sensory acceptability	[60]
Peach by-product	Muffins	- Fat substitute - Enhance Nutritional value (dietary fiber) - Reduce the caloric value - Increase the hardness - Acceptable sensory evaluation	[60]
Raspberry and Cranberry by products	Muffins	- Healthy nutritional profiles. - Improvement of viscoelastic properties and texture profile	[113]

**Table 6:** Effects of some fruit by-products on intestinal function.

by-Product	Extract	Extraction Technique	Dose	Effect	References
Yellow passion fruit (*Passiflora edulis*) peel	Soluble dietary fibres	Enzymatic-gravimetric method	0.1, 1 and 10 mg/kg (oral pre-treatment)	- Reduction of ethanol-induced gastric lesions in 72.25%, 79.23% and 87.17% - Avoiding depletion of GSH levels in 52.20%, 41.91% and 50.33% - Preventing gastric wall mucus in 26.32%, 25.03% and 31.00%	[149]
			1 mg/kg (intraperitoneal route)	- Reduction of gastric lesion area in 72.56% - Preventing depletion of gastric wall mucus in 21.24% and the GSH levels in 40.81%	
Avocado (*Persea americana* Mill.) seeds	Ethyl acetate extract (SEAP)	Hydroalcoholic extraction with 70% ethylic alcohol	10, 35 and 75 mg/kg (oral gavage)	- Significant ulcer index (UI) (2.89 ± 1.75, 2.4 ± 1.24 and 1.51 ± 0.72 for 10, 35 and 75 (UI) mg/kg, respectively) compared to lansoprazole which is a classic proton pump inhibitor (3.53 ± 1.50 UI) - Treatment with SEAP showed 92% of protection, while lansoprazole treatment showed 81% of protection - Increase of mucus production by the stomach mucosal cells of about 2.38 ± 0.55 times compared to the control	[150]
Avocado (Persea Americana) leaves	Aqueous and methanolic extracts	- Water extraction (72 h) - Soxhlet (24 h/MeOH)	200 mg/kg (intraperitoneal injection)	Significant inhibition of histamine-stimulated acid secretion through the action on H2-receptors	[151]
Pomegranate (*Punica granatum*) peels	Ethanol extract	Ultrasonic extraction with 99% ethanol	500 mg/kg (Oral)	- Significant protection of stomach mucosal layer from gastric ulcer induced by ethanol. - Stomach mucosal layer protection was improved by the increase in the dose.	[152]
Orange (*Citrus sinensis* L.) peels	Flavonoids	Extraction with 80% aqueous methanol	100, 200 and 400 mg/kg	- Correction of the enhanced gastric lipid peroxidation - Reduction of TNF-α (tumor necrosis factor alpha) production - Reduction of gastric DNA fragmentation and gastric COX-2 (cyclooxygenase-2) expression	[153]
Apple (*Malus domestica* cv.) peels	Polyphenol-rich extract	Retention on absorber resin Sepabeads SP-850	150 and 300 mg/kg/day (Oral ingestion)	Avert the initial attachment of *Helicobacter pylori* to the antral mucosa and suppress inflammation	[154]
Apple (*Malus domestica*) pomace	Pectin	Hot water-acid extraction	25 mg/kg (oral)	- Prevention of the formation of gastric mucosa lesions. - Lining the surface of the stomach - Improving the resistance of the gastroduoden mucosa	[147]
Grape seeds	Proanthocyanidins	Commercial product	100 mg/kg	- Significant decrease in ulcer index and TBARS level (dose dependent) - decrease in ulcer index and gastric acidity in pylorus ligation models - Reduction in MDA (malondialdehyde) levels - Decrease in the elevated levels of nitrite/nitrate, which are involved in the formation of intestinal inflammation	[141]
Orange bagasse and passion fruit peels	Alcohol insoluble solids	Enzymatic extraction/Extraction with 80% aqueous EtOH (4°/1 h)	−	- Energy source for bacteria located in the distal colon - Could be considered as an agent for selective modulation of the gut microbiota	[138]
Acerola (*Malpighia emarginata*) by-product (dried skin, seeds and pulp residues)	Polyphenol-rich extract	Extraction with 70% aqueous methanol	−	- Decrease in NH_4_^+^ amount in the ascending colon. - Increase in short-chain fatty acids (SCFAs) in the colon three regions - Positive effect on the gut microbiota metabolism for the probiotic strain *Bifidobacterium longum* BB-46 when combined with acerola by-product	[155]
By-products of acerola (*Malpighia emarginata* D.C.), cashew (*Anacardium occidentale* L.) and guava (*Psidium guajava* L.) fruits	Phenolic compounds and dietary fibres	Freezing in liquid N2 and freeze-drying	400 mg/kg body weight (orogastric administration)	- Reduction of body weight (3.42%, 3.08% and 5.20%) in dyslipidaemic female rats - Decrease in faecal pH, liver fat, visceral fat and serum lipid levels - Increase in faecal moisture, faecal fat excretion, amounts of organic acids in faeces and faecal *Bifidobacterium* spp. and *Lactobacillus* spp. counts. - Protection of colon and liver from tissue damage (e.g., destruction of liver and colon cells and increased fat deposition in hepatocytes) induced by dyslipidaemic diet.	[156]
